# A rational approach for cancer stem-like cell isolation and characterization using CD44 and prominin-1(CD133) as selection markers

**DOI:** 10.18632/oncotarget.12100

**Published:** 2016-09-17

**Authors:** Yi-Jen Lee, Chang-Cheng Wu, Jhy-Wei Li, Chien-Chih Ou, Shih-Chung Hsu, Hsiu-Hsueh Tseng, Ming-Ching Kao, Jah-Yao Liu

**Affiliations:** ^1^ Graduate Institute of Medical Sciences, National Defense Medical Center, Taipei, Taiwan; ^2^ Chief of Obstetrics and Gynecology, Tri-Service General Hospital Penghu Branch, Penghu, Taiwan; ^3^ Chief of Pathology, Da-Chien General Hospital, Miaoli, Taiwan; ^4^ Department of Rehabilitation science, Jente Junior College of Medicine, Nursing and Management, Miaoli, Taiwan; ^5^ Obstetrics and Gynecology, Tri-Service General Hospital, Taipei, Taiwan; ^6^ Medical Care and Management, Kang-Ning Junior College, Taipei, Taiwan; ^7^ Graduate Institute of Life Sciences, National Defense Medical Center, Taipei, Taiwan; ^8^ Department of Biological Science and Technology, China Medical University, Taichung, Taiwan; ^9^ Department of Biochemistry, National Defense Medical Center, Taipei, Taiwan; ^10^ Department of Obstetrics and Gynecology, National Defense Medical Center, Taipei, Taiwan; ^11^ Tri-Service General Hospital, National Defense Medical Center, Taipei, Taiwan

**Keywords:** cancer stem-like cells (CSLC), intraperitoneal enrichment, CD44 and CD133, SKOV3.PX1_133^+^44^+^, OVCAR3.PX1_133^+^44^+^

## Abstract

The availability of adequate cancer stem cells or cancer stem-like cell (CSC) is important in cancer study. From ovarian cancer cell lines, SKOV3 and OVCAR3, we induced peritoneal ascites tumors in immunodeficient mice. Among the cells (SKOV3.PX1 and OVCAR3.PX1) from those tumors, we sorted both CD44 and CD133 positive cells (SKOV3.PX1_133^+^44^+^, OVCAR3.PX1_133^+^44^+^), which manifest the characteristics of self-renewal, multi-lineage differentiation, chemoresistance and tumorigenicity, those of cancer stem-like cells (CSLC). Intraperitoneal transplantation of these CD44 and CD133 positive cells resulted in poorer survival in the engrafted animals. Clinically, increased CD133 expression was found in moderately and poorly differentiated (grade II and III) ovarian serous cystadenocarcinomas. The ascites tumor cells from human ovarian cancers demonstrated more CD133 and CD44 expressions than those from primary ovarian or metastatic tumors and confer tumorigenicity in immunodeficient mice. Compared to their parental cells, the SKOV3.PX1_133^+^44^+^ and OVCAR3.PX1_133^+^44^+^ cells uniquely expressed 5 CD markers (CD97, CD104, CD107a, CD121a, and CD125). Among these markers, CD97, CD104, CD107a, and CD121a are significantly more expressed in the CD133+ and CD44+ double positive cells of human ovarian ascites tumor cells (Ascites_133^+^44^+^) than those from primary ovarian or metastatic tumors. The cancer stem-like cells were enriched from 3% to more than 70% after this manipulation. This intraperitoneal enrichment of cancer stem-like cells, from ovarian cancer cell lines or primary ovarian tumor, potentially provides an adequate amount of ovarian cancer stem-like cells for the ovarian cancer study and possibly benefits cancer therapy.

## INTRODUCTION

Cancer stem cells (CSCs) possess features that promote malignant potential, including cell differentiation, self-renewal, tumorigenicity, chemoresistance and metastasis [1]. Dick et al. first isolated CSCs from patients with acute myeloid leukemia, which have the ability to proliferate as hematopoietic stem cells and differentiate into leukemic cells [2, 3]. To date, CSCs have been isolated from solid breast, brain, lung, liver, mouth, ovary, prostate, and colon tumors. To identify breast CSCs, a subpopulation of primary cancer cells expressing markers of self-renewal and differentiation potential are isolated and tested for tumorigenicity in mouse xenograft models [4].

Bapat et al. isolated two clones of ovarian cancer stem-like cells (CSLC) capable of sphere formation from ascites cultures [5], whereas Zhang et al. used cell sorting to isolate CD44 and CD117 double-positive ovarian cancer-initiating cells from primary human tumors [6]. Using three human ovarian cancer cell lines and four clinical human primary ascites cell lines, Szotek et al. identified a verapamil-sensitive subpopulation that expressed breast cancer resistance protein 1 [7]. Regardless of the methods, either sphere formation or cell sorting, the yield of the cancer stem cells or cancer stem-like cells thus harvested is pretty low (less than 1%). Hence it takes tremendous efforts to collect an adequate amount of stem cells for study [5]. An easier method to enrich the cancer stem cells or cancer stem-like cells will facilitate the biological study of cancer stem cells.

In ovarian cancer cells SKOV3 and OVCAR3, originated from human ovarian cancer ascites cells, expression of either CD44, CD24 or CD133 markers is related to drug-resistance, a stem-like characteristics of cancer cells [8–10]. Yu et al. reported that cancer cells from ascites induced by SKOV3 cells possesses enhanced malignant characteristics, including a rapid growth rate, enhanced ability of colony formation, and a shortened survival of the host animals [11, 12]. The highly therapy-resistant OVCAR3 was used for studying the mechanisms of drug- and radiation-resistance. Owing to the above biological characteristics, SKOV3 and OVCAR3 cells were used in our study for the enrichment of the stem-like cells.

In the literature, CD44 and CD133 expressions were risk factors for ovarian cancer metastasis and poor survival. The ascites cells with high CD44 and CD133 expressions, from ovarian cancer patients, displays more potentials for self-renewal and long-term proliferation [7, 8, 13, 14]. CD44 expression activates nanog protein, a stemness marker, which enhances chemoresistance in breast and ovarian cancer cells. Hence we attempted to enrich the cancer stem-like cell population by using CD 44, CD133 as selection markers.

In advanced cancer, proliferating CSC-like cells determine disease progression, prognosis, and chemoresistance [15]. We hypothesized that a small subpopulation of CSC-like malignant progenitor cells could be enriched from ascites induced in immunodeficient mice via the intraperitoneal inoculation of ovarian cancer cells (SKOV3 and OVCAR3 cells). These ascites CD133^+^44^+^ cells exhibited similar proliferating cancer stemloids characteristics, such as proliferation, chemoresistance, and tumor progression [16]. Our stem-like cell enrichment method could facilitate biological studies of ovarian CSCs, target-drug discovery and studies in other cancer types.

## RESULTS

### SKOV-3 and OVCAR3 ascites cells exhibited malignant tumor progenitor cells characteristics

**Figure 1:**
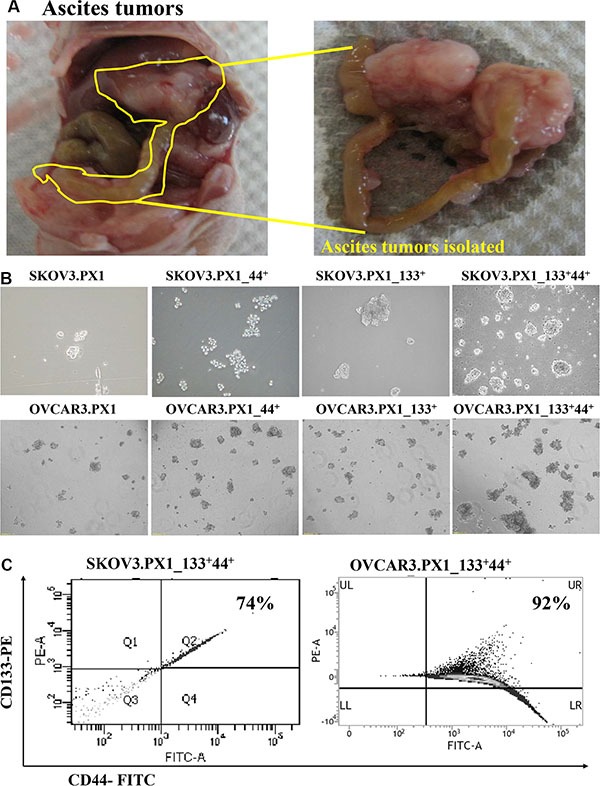
SKOV3 and OVCAR3 cells from tumor ascites and SKOV3.PX1_133^+^44^+^ and OVCAR3.PX1_133^+^44^+^ cells exhibit characteristics of malignant tumor progenitor cells (**A**) SKOV3 and OVCAR3 ascites tumors. SKOV3 and OVCAR3 cells were injected intraperitoneally into nude mice, yielding abdominal ascites and peritoneal tumor formation. Most tumors grew on the pancreas and mesentery. (**B**) CD133^+^/CD44^+^ ascites cells exhibited increased sphere formation. Four cell subsets were grown in sphere formation cultures with CSC media in ultra-low-attachment dishes. Cells were photographed at 100× magnification. (**C**) CD133+ and CD44+ expression in SKOV3.PX1_133^+^44^+^ and OVCAR3.PX1_133^+^44^+^ cells.

In this study, peritoneal cavity tumors mostly grew on the pancreas and mesentery after intraperitoneal inoculation of SKOV3 and OVCAR3 cells (Figure 1A). The cells from ovarian ascites tumors (SKOV3.PX1 and OVCAR3.PX1 cells) were cultured in DMEM/F12 medium supplemented with 10% FBS. We sorted SKOV3.PX1 and OVCAR3.PX1 cells based on the expression of CD44 and CD133 and cultured the four types of cells (PX1, PX1_44^+^, PX1_133^+^ and PX1_133^+^44^+^ cells) in CSC culture media.

All these types of cells formed spheres indicating the self-renewal capability of the SKOV3.PX1 and OVCAR3.PX1 cells (Figure 1B). The SKOV3.PX1_133^+^44^+^ and OVCAR3.PX1_133^+^44^+^ cells were most capable of rapid proliferation and sphere formation when grown in these ultra-low attachment dishes. After 3–5 passages, 74% and 92% of SKOV3.PX1_133^+^44^+^ and OVCAR3.PX1_133^+^44^+^ cells respectively sustained CD133 and CD44 expression (Figure 1C).

### CD133 and CD44 expressions of ovarian ascites cells possessed stem-like characteristics

**Figure 2:**
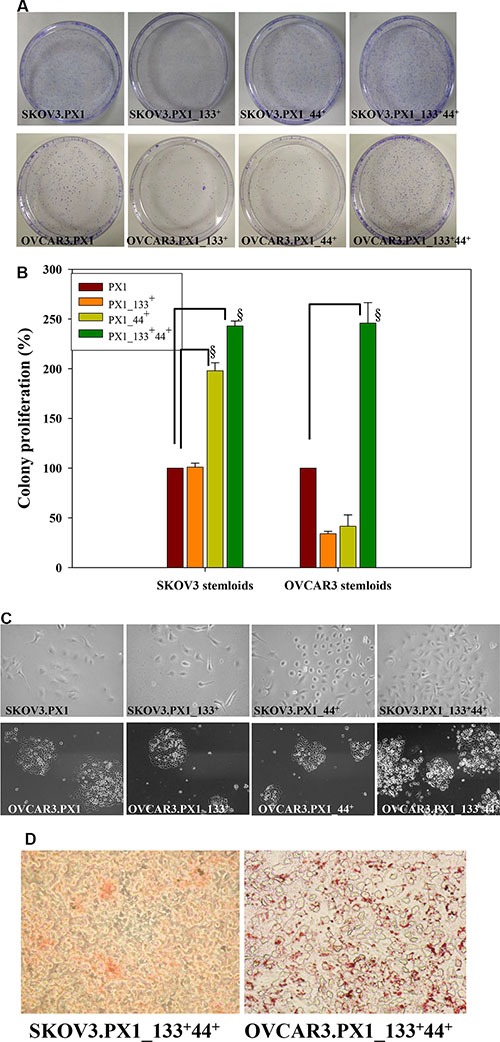
Stemness characteristics of SKOV3.PX1_133^+^44^+^ and OVCAR3.PX1_133^+^44^+^ cells (**A**) SKOV3.PX1_133^+^44^+^ and OVCAR3.PX1_133^+^44^+^ cells exhibited extensive colony formation in low-serum medium. (**B**) Cell proliferation rates (bars = means ± standard deviations [SD]) relative to the rates of SKOV3.PX1 and OVCAR3.PX1 in 1% FBS (control). Proliferation ratios of four cell subsets in different culture media were calculated as follows: [(treatment cell number/control cell number) × 100%]. A *t*-test was used to calculate *p* values; ^§^*p* < 0.0005. (**C**) SKOV3.PX1_133^+^44^+^ and OVCAR3.PX1_133^+^44^+^ cells proliferated more rapidly than other cells in low-serum medium. Four cell subsets were seeded into 6-cm fibronectin-coated dishes, cultured for 8 days, and photographed at 100× magnification. (**D**) SKOV3.PX1_133^+^44^+^ and OVCAR3.PX1_133^+^44^+^ cells differentiated into adipocytes (Oil red O staining). Cells were photographed at 100× magnification.

Self-renewal, clonogenic expansion, multilineage differentiation, enhanced tumorigenic potential, and chemoresistance are key characteristics of CSCs [17]. A fibronectin-rich niche supports mammary gland development and tumorigenesis; it augments cell proliferation [18]. To examine if PX1 subpopulations had the ability to proliferate under minimal environments, we tested the colony forming ability of SKOV3.PX1_133^+^44^+^ and OVCAR3.PX1_133^+^44^+^ cells in limited-serum medium in fibronectin-coated dishes. SKOV3.PX1_133^+^44^+^ and OVCAR3.PX1_133^+^44^+^ cells formed a number of colonies in media supplemented with 1% FBS, whereas the other types of cells formed few colonies (Figure 2A). Furthermore, SKOV3.PX1_133^+^44^+^ and OVCAR3.PX1_133^+^44^+^ cells proliferated more rapidly than the other types of cells in a 1% FBS culture media. Under these conditions, SKOV3.PX1_44^+^ cells proliferated faster than SKOV3.PX1_133^+^ cells (Figure 2B–2C). CD44 expression rather than CD133 expression conferred greater proliferative potential and self-renewal capacity.

We analyzed the differentiation potential of two cancer stem-like cells and found that when induced, cancer stem-like cells differentiated into adipocytes (Figure 2D). These results demonstrated that SKOV3.PX1_133^+^44^+^ and OVCAR3.PX1_133^+^44^+^ cells, similar to mesenchymal stem cells, possess the capacity for differentiation into adipocytes.

In summary, the CD133^+^/CD44^+^ subpopulations of SKOV3.PX1 and OVCAR3.PX1 possess identical self-renewal, clonogenic expansion, and differentiation capabilities.

### Chemoresistance capability of SKOV3.PX1_133+44+ and OVCAR3.PX1_133+44+ cells

The IC_50_ of paclitaxel for SKOV3.PX1 and SKOV3.PX1_133^+^44^+^ cells were 82 nM and 1000 nM (12-fold) respectively. The SKOV3.PX1_133^+^44^+^ cells exhibit greater drug resistance than SKOV3 and SKOV3.PX1 cells. In addition, the SKOV3.PX1_133^+^44^+^ cells showed resistance to cisplatin, doxorubicin and paclitaxel with an IC_50_ higher (> 20-fold, > 20-fold and 80-fold) than those for SKOV3 cells. Similarly, the OVCAR3.PX1_133^+^44^+^ cells showed resistance to cisplatin, doxorubicin and paclitaxel with an IC_50_ higher (2.5-fold, 2.5-fold and 80-fold) than those for OVCAR3 cells (Table 1).

**Table 1:** Chemoresistance of SKOV3.PX1_133^+^44^+^ and OVCAR3.PX1_133^+^44^+^ cells

Cells	IC_50_ (μM)		
	Cisplatin	Doxorubicin	Paclitaxel
SKOV3	5 ± 2	1.7 ± 0.8	0.018 ± 0.01
OVCAR3	69 ± 1.2	67 ± 2.4	0.012 ± 0.01
SKOV3.PX1_133^+^44^+^	> 100	> 100	> 1
OVCAR3.PX1_133^+^44^+^	> 150	> 150	> 1

Suspensions of four cell subsets 0.18 ml (6 × 10^4^/ml) were seeded into microtiter plates and incubated overnight in growth medium. Subsequently, cells were treated with chemotherapeutic drugs at various concentrations for 3 days. Finally, cells were treated with MTT, and the absorbance at 545 nm was measured in each well with a microplate reader (Molecular Devices, Eugene, OR, USA; reference: absorbance at 690 nm). Growth inhibition was estimated as follows: [1-(optical density of drug treatment/optical density of control)] × 100%. The IC_50_ (50% growth inhibition) for a particular drug was estimated from a plot of drug concentration versus percentage growth inhibition.

**Figure 3:**
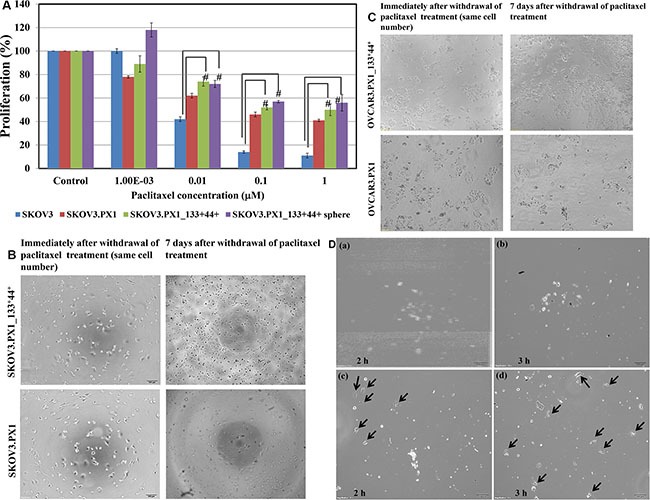
Chemoresistance and chemotaxis of SKOV3.PX1_133^+^44^+^ and OVCAR3.PX1_133^+^44^+^ cells (**A**) Paclitaxel cytotoxicity among SKOV3, SKOV3.PX1, SKOV3.PX1_133^+^44^+^, and SKOV3.PX1_133^+^44^+^ sphere cells. Sphere cells exhibited superior survival after paclitaxel treatment at various concentrations (*t*-test, ^#^*p* < 0.005). (**B**–**C**) SKOV3.PX1_133^+^44^+^ cells exhibited superior recovery after paclitaxel withdrawal. SKOV3.PX1_133^+^44^+^ cells exhibited better proliferation versus SKOV3.PX1 cells 7 days after paclitaxel withdrawal. Cells were photographed at 100× magnification. OVCAR3.PX1_133^+^44^+^ cells behaved similarly. (**D**) Chemotactic capability of SKOV3.PX1_133^+^44^+^ cells. A 100-μl aliquot of SKOV3.PX1 cells was added to the upper deck of each transwell, and conditioned media from SKOV3.PX1 or SKOV3.PX1_133^+^44^+^ cells was added to the lower decks. SKOV3.PX1 cells penetrated the transwell membranes and migrated to the lower decks after two hours (arrows: SKOV3.PX1 cells in lower decks panels a and b: SKOV3.PX1 conditioned medium at two and three hours; c and d: SKOV3.PX1_133^+^44^+^ conditioned medium at two and three hours). Cells were photographed at 100× magnification.

After paclitaxel treatment, the survival rates of each cell types in different culture conditions shown in Figure 3A. Proliferation reduced to 50 % when the drug concentration increased from 10 nM to 1000 nM. In addition, for SKOV3.PX1_133^+^44^+^ cells, those grown in sphere cultures (floating sphere) demonstrates more drug resistance than those grown in adherent cultures. Furthermore, 7 days after paclitaxel withdrawal, surviving SKOV3.PX1_133^+^44^+^ and OVCAR3.PX1_133^+^44^+^ cells re-grew, unlike SKOV3.PX1 cells (similar numbers of surviving cells after withdrawal of different paclitaxel concentrations; Figure 3B and Figure 3C).

### Chemotactic capability of SKOV3.PX1_133+44+ cells

For the chemotaxis experiments, 5 × 10^4^ SKOV3.PX1 cells were added to the upper decks of the transwells; the condition media of SKOV3.PX1 and SKOV3.PX1_133^+^44^+^ cells were added respectively to the lower decks. The condition media of SKOV3.PX1_133^+^44^+^ attracted more SKOV3.PX1 cells migration, in 2 h- and 3 h-periods (Figure 3D(c-d), black arrows). This demonstrated that the SKOV3.PX1_133^+^44^+^ cells secreted more factors to facilitate cancer cells migration.

### Tumor-initiating ability of CD133+CD44+ CSC-like cells from ascites

**Figure 4:**
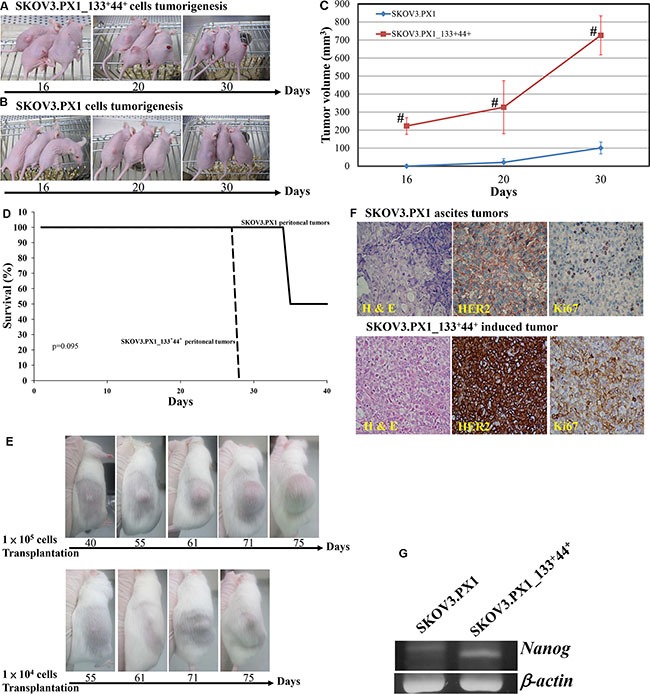
Tumorigenicity of SKOV3.PX1 and SKOV3.PX1_133^+^44^+^ cells To induce tumor formation, 5 × 10^5^ SKOV3.PX1_133^+^44^+^ cells (**A**) or SKOV3.PX1 cells (**B**) were transplanted into the dorsa of three nude female mice. At day 16, solid tumors formed from SKOV3.PX1_133^+^44^+^ cells (mean tumor volume = 223 ± 46 mm^3^); SKOV3.PX1 cells had not form tumors. On day 30, solid tumors had grown in two of three SKOV3.PX1-transplanted animals (mean tumor volume = 101 ± 33 mm^3^ vs. 726 ± 108 mm^3^ in SKOV3.PX1_133^+^44^+^-transplanted animals). (**C**) SKOV3.PX1 and SKOV3.PX1_133^+^44^+^ tumor growth curves (*t*-test, ^#^*p* < 0.005). (**D**) Survival curves of mice following the intraperitoneal transplantation of SKOV3.PX1 and SKOV3.PX1_133^+^44^+^ cells. (**E**) Tumor initiation abilities of SKOV3.PX1_133^+^44^+^ cells. Either 1 × 10^5^ or 1 × 10^4^ of SKOV3.PX1_133^+^44^+^ cells were transplanted into the dorsa of SCID/NOD female mice; tumors were initially detected in all animals on day 40 or day 55, respectively. (**F**) Enhanced HER2 and Ki-67 expression in SKOV3.PX1_133^+^44^+^-induced tumors. Compared with SKOV3.PX1 tumors, SKOV3.PX1_133^+^44^+^ tumors exhibited strongly elevated HER2 and Ki67 expression (hematoxylin and eosin staining; HER2 and Ki67 immunohistochemistry). (**G**) SKOV3.PX1_133^+^44^+^ cells express the stemness gene *nanog*. *Nanog* gene expression in SKOV3.PX1 and SKOV3.PX1_133+44+ cells after real-time PCR amplification. Beta-actin was used as a housekeeping gene. PCR for *nanog* and *beta-actin* yielded 158 bp and 220 bp products, respectively.

For *in vivo* tumorigenicity studies, 5 × 10^5^ SKOV3.PX1 or SKOV3.PX1_133^+^44^+^ cells were each transplanted subcutaneously into the dorsum of female nude mice. By day 16, solid tumors with an average volume of 223 ± 46 mm^3^ grew in all SKOV3.PX1_133^+^44^+^-transplanted mice (Figure 4A); while SKOV3.PX1 cells did not induce tumor formation yet (Figure 4B). In addition, subcutaneous transplantation of SKOV3.PX1_133^+^44^+^ tumors grew rapidly (Figure 4C). Intraperitoneal injection of SKOV3.PX1_133^+^44^+^ cells were associated with poor survival of the animals (Figure 4D). Next, 1 × 10^5^ or 1 × 10^4^ SKOV3.PX1_133^+^44^+^ cells were injected subcutaneously into the dorsum of each SCID/NOD female mouse. Solid tumors developed in all mice after 40 and 55 days, respectively (Figure 4E), demonstrating the tumorigenicity of SKOV3.PX1_133^+^44^+^ cells.

We evaluated expression of the proliferation marker Ki-67 in SKOV3.PX1 and SKOV3.PX1_133^+^44^+^-induced tumors by scoring immunostained tumor sections. As shown in Figure 4F, Ki-67 proliferation index evaluation revealed a significantly higher percentage of labeled cells in SKOV3.PX1_133^+^44^+^-induced tumors relative to SKOV3.PX1-induced tumors (80%–90% versus 10%–15%).

### Tumors induced by SKOV3.PX1_133+44+ cells overexpress HER2

As shown by Ki-67 immunohistochemistry (IHC) staining, pancreatic and mesenteric ovarian tumors proliferated rapidly. According to the Hercep Test scoring system [19], HER2 expression in ovarian ascites cells scored 3. By IHC staining, enhanced overexpression of HER2 was found in the tumors induced by the SKOV3.PX1_133^+^44^+^ cells than those by the SKOV3.PX1 cells (Figure 4F).

In summary, SKOV3.PX1_133^+^44^+^ and OVCAR3.PX1_133^+^44^+^ cells demonstrated cancer stem-like cell characteristics. These ascites tumor derived cancer stem-like cells behaved similarly to the clinically advanced ovarian cancer, exhibiting oncogene overexpression, chemoresistance, tumorigenicity, and rapid metastasis.

### Human primary-ovarian-cancer ascites cells are tumorigenic

**Figure 5:**
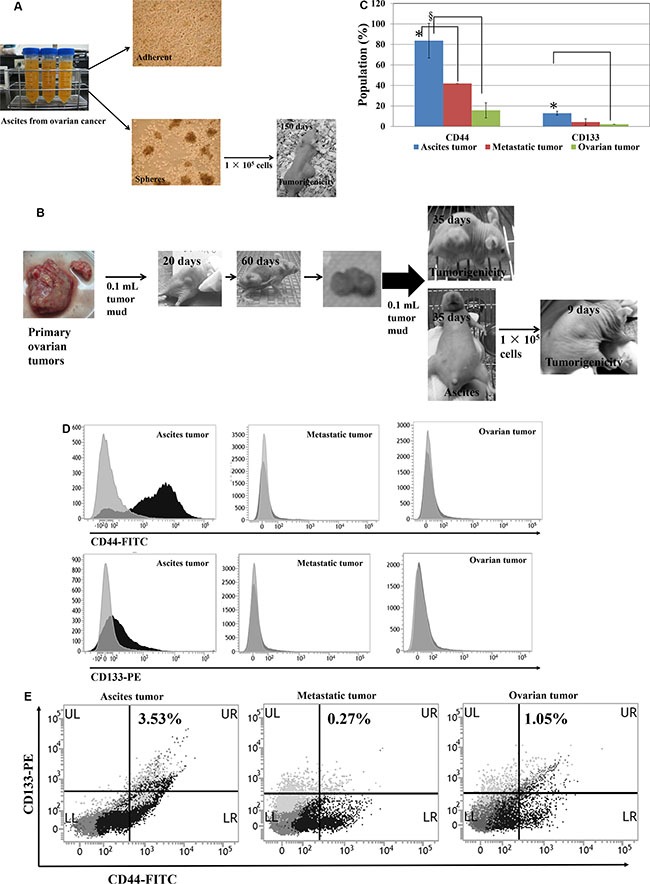
CD133^+^/CD44^+^ subpopulations and tumorigenicity of human ovarian ascites cells and ovarian tumors (**A**) Human primary ovarian ascites cells (HOVCA_AS) exhibit tumorigenicity. HOVCA_AS cells were grown from the ascites of a patient with primary ovarian epithelial cancer (images: 100× magnification) and formed spheres when cultured in ultra-low attachment culture dishes (Corning, Corning, NY, USA). Transplantation of 1 × 10^5^ HOVCA_AS cells to the dorsa of nude female mice led to tumor formation. (**B**) Repeated *in vivo* transplantation enhanced the tumorigenicity of human primary ovarian tumors. (**C**–**D**) CD133^+^ and CD44^+^ expressions in clinical primary-ovarian-cancer specimens and normal ovarian cells. CD44 (FITC) and CD133 (PE) expression in primary-ovarian-cancer ascites cells (*N* = 5), metastatic tumors (*N* = 3), ovarian tumors (*N* = 5), and normal ovarian cells (*N* = 7) was determined using flow cytometry (BD Biosciences, San Jose, CA, USA; fluorescent markers: FITC and PE, respectively; *t*-test, *p < 0.05; ^§^*p* < 0.0005). (**E**) Representative double CD44-FITC and CD133-PE staining of a clinical primary-ovarian-cancer specimen.

Human primary-ovarian-cancer ascites cells and tumors were collected from patients with advanced ovarian cancer and ascites. Cells from cancer-cell spheres were subcultured to generate HOVCA_AS cells. Tumor formation was observed 150 days after implanting 1 × 10^5 HOVCA_AS cells^ into the dorsum of each female nude mouse (Figure 5A).

Next, human primary-ovarian-cancer tumors were homogenized, and a 0.1-mL aliquot of homogenate was subcutaneously transplanted to the dorsum each female nude mouse. Solid tumor formation was observed in 20 days and tumors grew to an average size of over 1000 mm^3^ in 60 days. The homogenates from these tumors were injected intraperitoneally into nude mice. Ascites formation was observed after 35 days, and ascites tumor cells were collected and injected into the dorsa of nude mice. Notably, these ascites tumor cells formed tumors in 9 days compared to 20 days in those cells from primary tumor (Figure 5B). Tumorigenicity of the tumor cells from human primary ovarian cancers was enhanced after intraperitoneally injection into nude mice.

### Human ovarian cancer ascites cells expressed more CD133 and CD44

Aggressive CSC populations evolve from primary CSCs through the acquisition of successive mutations [20] with late-stage tumors containing higher percentage of CSC populations [21]. Our results show that human ovarian cancer ascites cells significantly expressed more CD44-FITC (*p* < 0.005) and CD133-PE (*p* < 0.05), compared with primary ovarian tumors (Figure 5C and 5D). Furthermore, they have a higher percentage of double positive (CD133^+^, CD44^+^) cells than the primary tumors (Figure 5E).

### Candidate cluster of differentiation (CD) markers of cancer stem-like cells

**Figure 6:**
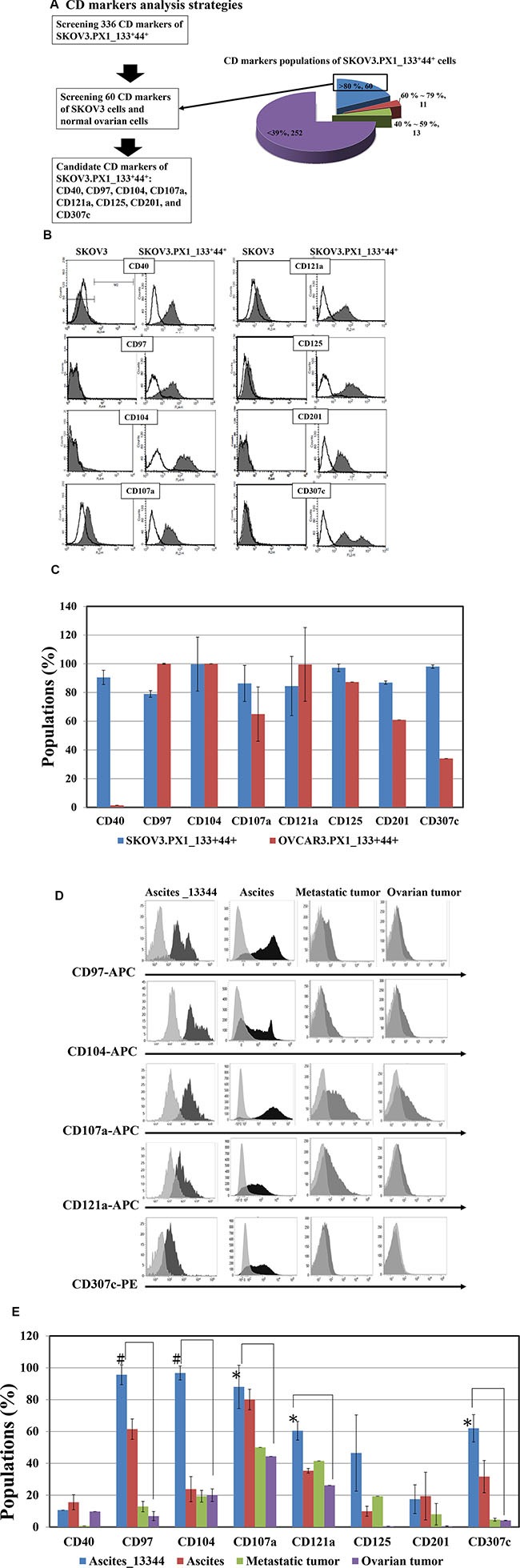
Candidate CD markers of ovarian SKOV3.PX1_133^+^44^+^ CSC-like cells (**A**) CD marker analysis strategy. Surface marker expression in normal ovarian, SKOV3, and SKOV3.PX1_133^+^44^+^ cells was analyzed by flow cytometry. A total of 336 surface markers were evaluated in SKOV3.PX1_133^+^44^+^ cells; of these, 60 CD markers were strongly expressed (> 80%). Among these 60 markers, eight (CD40, CD97, CD104, CD107a, CD121a, CD125, CD201, and CD307c) exhibited differential strong expression in SKOV3.PX1_133^+^44^+^ cells versus parental SKOV3 and normal ovarian cells. (**B**) Fluorescently determined expression of these eight markers in SKOV3 and SKOV3.PX1_133^+^44^+^ cells. (**C**) Expression of these eight markers in OVCAR3.PX1_133^+^44^+^ and SKOV3.PX1_133^+^44^+^ cells. Marker expression was analyzed by flow cytometry. (**D**) Expression of the eight markers in clinical Ascites_133^+^44^+^ cells and primary-ovarian-cancer specimens. Fluorescence expression levels were determined by flow cytometry. (**E**) Populations of clinical primary-ovarian-cancer specimens and normal ovarian cells that expressed the eight markers, determined via flow cytometry with FITC- and PE-conjugated antibodies against CD44 and CD133, respectively; Ascites_133^+^44^+^ (*N* = 3), primary-ovarian-cancer ascites (*N* = 5), metastatic tumor (*N* = 3), ovarian tumor (*N* = 5), and normal ovarian cells (*N* = 7) were analyzed (*t*-test, *p < 0.05; ^#^*p* < 0.005).

Of the 336 surface markers tested, 60 CD markers were expressed in over 80% of the SKOV3.PX1_133^+^44^+^ cells (Figure 6A). Among these 60 CD markers, 29 CD markers (CD109, CD138, CD317, CD58, CD136, CD266, CD47, CD95, HER2, CD277, CD324, CD344, CD239, CD318, CD213a1, CD112, CD164, CD49a, CD71, CD166, CD171, CD24, CD326, CD70, EGFR, CD98, CD49f, stage-specific embryonic antigen-4 (SSEA-4) and CD340) expressed in both SKOV3 cells and SKOV3.PX1_133^+^44^+^ cells, eight markers (CD40, CD97, CD104, CD107a, CD121a, CD125, CD201, and CD307c) were overexpressed only in SKOV3.PX1_133^+^44^+^ cells (Figure 6B). We evaluated the expression of these markers in OVCAR3.PX1_133^+^44^+^ cells via flow cytometry. CD97, CD104, CD107a, CD121a, and CD125 all exhibited > 50% fluorescence in SKOV3.PX1_133^+^44^+^ and OVCAR3.PX1_133^+^44^+^ cells (Figure 6C). The five markers CD97, CD104, CD107a, CD121a, and CD125, expressed in SKOV3.PX1_133^+^44^+^ and OVCAR3.PX1_133^+^44^+^ cells, are related to activation of the ITGB4–EGFR pathway which eventually promote cell proliferation [22].

### CD marker expression in human ascites CD133+CD44+ CSC-like cells

Using flow cytometry, we evaluated CD133 and CD44 expression in human primary-ovarian-cancer specimens (ascites cells, metastatic tumors, ovarian tumors, and normal ovary). CD133 and CD44 expression, alone or together, was the highest in human primary-ovarian-cancer ascites cells. Furthermore, CD97, CD104, CD107a, CD121a, and CD307c were significantly more expressed in Ascites_133^+^44^+^ cells than in ovarian or metastatic ovarian tumors (*p* < 0.005, *p* < 0.05, respectively; Figure 6D, 6E).

### Overexpressed markers in Ascites_133+44+ cells possess ITGB4–EGFR pathway activating functions

CD97 enhances cell migration, activates proteolytic matrix metalloproteinases, and increases interleukin (IL)-8 secretion [23] and may be a target with which to modulate cancer progression [24, 25]. CD104 [integrin beta 4 (ITGB4)] mediates cell-matrix or cell-cell adhesion, enhances cell growth and signaling, and thus plays a pivotal role in cancer invasion [26]. CD107a (lysosome-associated membrane glycoprotein 1), higher expressed in metastatic ovarian neoplasia, correlates with the metastatic potential [27]. CD121a (IL-1 receptor, type I) activates PIK3R1 and MYD88. The activation of TLR4/MyD88/NF-κB signaling enhances aggressive tumor phenotype with poorer clinical outcome in epithelial ovarian cancer patients [28–30]. CD307c (Fc receptor-like 5) is differentially expressed in cancer cells. CD307c-conjugated anti-cancer drugs are effective against multiple myeloma *in vitro* and *in vivo* [31]. Ascites_133^+^44^+^, SKOV3.PX1_133^+^44^+^, and OVCAR3.PX1_133^+^44^+^ cells similarly expressed four of the five ITGB4–EGFR pathway activation markers.

### CD133 expression correlated with a high grade of cell differentiation in human ovarian serous cystadenocarcinoma

**Figure 7:**
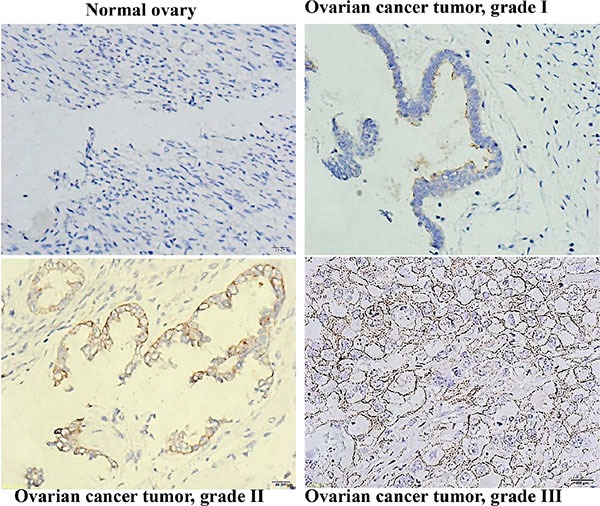
CD133 expression correlated with a high differentiation grade in human ovarian serous cystadenocarcinomas Immunohistochemical analysis of CD133 expression in human ovarian serous cystadenocarcinoma tissues; 400× magnification.

CD133 has been identified as a marker of enhanced proliferative potential in primary ovarian tumors [32]. We analyzed CD133 expression in 52 ovarian serous cystadenocarcinomas, stratified by grade of cell differentiation as the followings: grade I (10 samples, 19%), grade II (15 samples, 29%), and grade III (27 samples, 52%). Table 2 shows the correlation of CD133 expression with tumor grade. The mean score of CD133 expression of grade III tumors was significantly higher than that of grade I tumors (1.59 vs. 1.00; *p* < 0.05 (Figures in Supplementary Data S1). In normal ovarian tissues, CD133 expression was either absent or barely visible (Figure 7).

**Table 2:** CD133 expression in human ovarian serous cystadenocarcinoma tissues

Score Grade N*	CD133 expression score			Score Means^*1^, *p* value^*2^
	1	2	3	
I (10)	9	1	0	1.10
II (15)	9	4	2	1.53
III (27)	14	10	3	1.59 (*p* < 0.05)

Immunohistochemical analysis of CD133 expression in human ovarian serous cystadenocarcinoma tissues. CD133 expression correlated with tumor grade; specifically, a significant difference was observed between grades III and I.

*1: (1 × number of score 1) + (2 × number of score 2) + (3 × number of score 3)/total number of grade I or II or III.

*2: data are presented as mean CD133 scores ± SDs.

## DISCUSSION

Intraperitoneal inoculation of human ovarian cancer cell lines in immunodeficient mice (tumor ascites animal model), we identified the SKOV3.PX1_133^+^44^+^ and OVCAR3.PX1_133^+^44^+^ cells that exhibited cancer stem-like characteristics, including self-renewal, multilineage differentiation, tumorigenicity, and chemoresistance. This procedure enriched the subpopulation of cancer stem-like cells. These similar phenomena apply to cells from human ovarian cancers.

CD133 is an 866-amino-acid single-chain transmembrane glycoprotein (molecular weight 120 kDa). It is expressed in CSCs isolated from brain, prostatic, pancreatic, colonic, and ovarian epithelial tumors [33]. Previous studies have shown that CD133 is expressed in primary tumors and that CD133-positive cells had high proliferative potential and exhibited clonogenic expansion [32]. CD44 is expressed in breast, prostate, colon, and ovarian tumors [34]. Higher CD44 expression is found in cancer stem cell population and confers chemoresistance [35]. CD44 expression enhances stemness characteristics, include differentiation, spheroids formation, and tumorigenicity.

In SKOV3 and OVCAR3 cells, about 90 % of cells express CD44 and 0.7 % of cells express CD133. And less than 1% expresses both CD133 and CD44. This finding is consistent with the fact that cancer stem-like cells or cancer stem cells are a small population of cancer cells in the malignant tumors. Clinically, 80 to 90 % of ascites cancer cells from our ovarian cancer patients expressed CD 44, 5 to 15 % expressed CD133, and only 3 % expressed both CD44 and CD133. These are all significantly higher than those in primary ovarian tumors. The expression of CD133^+^/CD44^+^, mostly expressed in the cancer stem-like cells, are similar in SKOV3 or OVCAR3 induced cells and the ascites cancer cells from ovarian cancer patients. In our studies, primary ovarian cancer ascites cells easily formed spheres in Ultra Low Attachment Culture Dishes (Corning) in 3 days. Furthermore, when transplanted subcutaneously into nude mice, these primary ovarian cancer ascites cells formed solid tumor, show tumorigenicity potential. Enhanced tumorigenicity was shown after intraperitoneal inoculation of these cells in immunodeficient mice.

In ovarian cancer, CD44^+^/MYD88^+^expression denotes stem-like property and chemoresistance [35]. Through the bioinformatics, the eight CD markers expressed in SKOV3.PX1_133^+^44^+^ cells involve TRAF6 activation, in which the downstream signaling pathway involves MYD88 and IRAK activation. The five CD markers, CD97, CD104, CD107a, CD121a, and CD125, uniquely expressed in SKOV3.PX1_133^+^44^+^ cells and OVCAR3.PX1_133^+^44^+^ cells, play a role in EGFR regulation. In the results from our ovarian cancer patients, among these markers, CD97, CD104, CD107a, and CD121a are significantly more expressed in the CD133 and CD44 double-positive ascites cells (Ascites_133^+^44^+^ cells) than in ovarian or metastatic tumors. These ovarian cancer stem-like cells, SKOV3.PX1_133^+^44^+^ cells and OVCAR3.PX1_133^+^44^+^ cells, behave similarly to the CD133 and CD44 double-positive ascites cells (Ascites_133^+^44^+^ cells), which manifested in late-stage ovarian cancers. This intraperitoneal enrichment of cancer stem-like cells, from ovarian cancer cell lines or primary ovarian tumor, potentially provides an adequate amount of ovarian cancer stem-like cells for the study of ovarian cancer.

We investigated whether the observed enhanced chemoresistance of SKOV3.PX1_133^+^44^+^ cells was related to *MDR1* gene expression. The p170 protein encoded by the *MDR1* gene acts as a pump to lower the intracellular drug concentrations. Verapamil, a calcium channel blocker, inhibits the p170 protein pump and reverses the *MDR1* induced chemoresistance [36]. In our study, chemotherapy with cisplatin was used to treat SKOV3.PX1_133^+^44^+^ cells. However, our results demonstrated that verapamil cannot significantly potentiate the growth inhibition by cisplatin, suggesting that the chemoresistance of SKOV3.PX1_133^+^44^+^ cells is not mediated via *MDR1* gene expression.

Both SKOV3.PX1_133^+^44^+^ and SKOV3.PX1_133^+^ cells expressed drastically higher levels of HER2 than their parental cells (data not shown). HER2 overexpression is known to be related to chemoresistance, metastasis, and relapse in breast, gastric, and ovarian cancers. Overexpression of epithelial mucin (MUC4), regulated by HER2, is known to induce and enhance ovarian CSC proliferation. MUC4 overexpression is also associated with CD133 expression. The overexpression of HER2 illustrates the cancer-stem like characteristics of the SKOV3.PX1_133^+^44^+^ cells.

Furthermore, we found that 1 × 10^4^ SKOV3.PX1_133^+^44^+^ cells could not form solid tumors in *nude* mice (less immunocompromised compared to SCID/NOD mice) over a period of 3 months when injected subcutaneously, while intraperitoneal injection caused tumor and ascites formation and death of the animals. As shown in our tumor ascites model, the intraperitoneal injection augmented the cancer-stem like cells characteristics and caused the death of all the animals thus treated. This result further illustrates the possible intraperitoneal enrichment of cancer stem-like cells in the animals inoculated intraperitoneally.

The enrichment of cancer stem-like cells we have developed can benefit cancer study and potentially help the treatment of ovarian cancer. Hopefully it can be applied to other cancer types.

## MATERIALS AND METHODS

### Cell culture

The SKOV3 (ATCC HTB-77) and OVCAR3 (BCRC 60551) ovarian cancer cell lines respectively obtained from ATCC and BCRC and were maintained in Dulbecco's modified Eagle's medium (DMEM)/F12 medium (Gibco, Grand Island, NY) supplemented with 10% fetal bovine serum (FBS). Cells were incubated at 37°C in a humidified incubator equilibrated with 5% CO_2_.

For sphere growth, those sorted cells were cultured in serum-free DMEM/F12 medium supplemented with 10 ng/ml human recombinant epidermal growth factor (EGF; Invitrogen, Carlsbad, USA), 10 ng/ml basic fibroblast growth factor (βFGF, Invitrogen), and 0.4% bovine serum albumin (BSA; Sigma-Aldrich) in ultra-low attachment plates (Corning^®^). The above culture medium was named CSLC culture medium. Culture medium was changed every 2 days after centrifuging the cultures at 300 g for 5 min to remove debris.

### Culture of primary human ovarian cancer ascites cells

All studies were approved by the Institutional Review Board (IRB) of the Tri-Services General Hospital, Taipei, Taiwan. Ascites were collected from patients with ovarian cancer. Cells were collected after centrifugation at 300 g at 4°C for 10 minutes, and removal of red blood cells (RBCs) by Histopaque-1077 (Sigma). Cells were maintained in DMEM/F12 medium (Gibco) supplemented with 10% FBS and incubated at 37°C in a humidified atmosphere equilibrated with 5% CO_2_. These cells were named HOVCA_AS cells. Spheres grown from these HOVCA_AS cells were maintained in stem-like cells culture medium.

### Primary human ovarian cancer specimens

Clinical ovarian cancer samples (ovarian tumors and metastatic tumors) were washed with normal saline, minced (< 5 mm in size) and disassociated with Accutase^TM^ (eBioscience) at 37°C for 30 minutes and centrifuged at 300 g at 4°C for 10 minutes. Disassociated cells were obtained after removing supernatant and resuspension in medium with 10% FBS supplement, and then filtered through 40 mm cell strainers (BD Falcon).

### Ovarian cancer ascites formation in immune incompetent mice

All animal experiments were approved by the Institutional Animal Care and Use Committee (IACUC) of the National Defense Medical Center, Taipei, Taiwan. Suspensions of SKOV3 cells at a density of 5 × 10^6^/0.2 ml in Dulbecco's phosphate buffered saline (DPBS) were injected intraperitoneally into five female nude mice. Mice were initially observed twice weekly for signs of ascites formation after tumor development — abdominal bloating, loss of subcutaneous fat, hunched posture, and decreased movement and then followed up every two days before sacrifice of the animals. The intraperitoneal tumors were removed after sacrifice of the mice. After sieving the minced tumor debris (BD strainer, 40 μm), the tumor cells were harvested and were cultured.

These tumor cells collected from these ascites cells were named SKOV3.PX1 and OVCAR3.PX1. These ascites cells were labeled with CD44-fluorescein isothiocyanate (FITC) and CD133-phycoerythrin (PE) respectively, and sorted for positive cells by FACSorter (BD). The positive cells, named SKOV3.PX1_133^+^44^+^ and OVCAR3.PX1_133^+^44^+^ were maintained in stem-like cells culture medium. These cells formed non-adherent spheres under these conditions. The culture medium was changed every 2 days after centrifuging at 300 g for 5 min to remove debris.

### Tumorigenicity

All animal experiments were approved by the IACUC of the National Defense Medical Center, Taipei, Taiwan. The SKOV3.PX1_133^+^44^+^ cell were collected after dispersing the cell spheres with Accutase^TM^ (Biolegend) and then washed with DPBS, and centrifuged at 300 g. Suspensions of 1 × 10^5^ or 1 × 10^4^ cells in 0.1 ml DPBS were injected subcutaneously into the right flank of SCID/NOD female mice under aseptic conditions. The tumor size was estimated by the products of the length and width of the subcutaneous tumors. The growth of these tumors was monitored for 3–6 months after injection.

### Chemoresistance assay

MTT (3-(4,5-dimethylthiazol-2-yl)-2,5-diphenyl-tetrazolium bromide) cytotoxicity assay was used to evaluate the growth inhibition induced by chemotherapeutic drugs in various cells. Drugs were dissolved in dimethyl sulfoxide (DMSO; Sigma, D8779) to prepare stock solutions and were serially diluted with DPBS before use. MTT assays were performed as described by Wang et al. [37]. Briefly, cell suspensions were prepared from the monolayer cultures, seeded into microtiter plates (Nunc), and incubated in growth medium overnight. Cells were then treated with chemotherapeutic drugs at various concentrations for 3 days. Next, cells were treated with MTT and the absorbance at 545 nm was measured with a microplate reader (microplate reader, Molecular Device) using absorbance at 690 nm as a reference. Growth inhibition was estimated according to the following formula: [1-(optical density of drug treatment/optical density of control)] × 100%. The IC_50_ (for 50% growth inhibition) for a particular drug was estimated using a plot of drug concentration versus percentage growth inhibition.

### Adhesion assay

Suspensions of 12,000 cells were seeded into a 6-cm dish coated with fibronectin and cultured in medium containing 1% and 5% FBS medium. The cultured cell types included SKOV3 and OVCAR3, CD44 positive cells, CD133 positive cells, and CD44 & CD133 double positive cells. Growth and adhesion were assessed by counting the number of cells by hemocytometer, 8 days after seeding.

### Colony formation assay

Suspensions of 12,000 cells were plated onto fibronectin-coated 6-cm dishes and cultured in medium containing 1% and 5% FBS. Thus, the cultured cell types included SKOV3 and OVCAR3, CD44 positive cells, CD133 positive cells, and CD44 & CD133 double positive cells. Colony formation was monitored between days 1 to 8 after seeding, and the colonies were stained using crystal violet on the eighth day.

### Adipogenic differentiation assay

The SKOV3.PX1_133^+^44^+^ and OVCAR3.PX1_133^+^44^+^cells (1 × 10^5^) were seeded into 6-cm dishes and cultured in induction medium. The adipogenic induction medium consisted of 5% FBS, 1 μM dexamethasone, 0.5 mM 3-isobutyl-1-methylxanthine (IBMX), 5 μg/ml insulin, and 60 μM indomethacin. The culture medium was changed every 3 to 4 days. After 10 days of differentiation, cells were fixed with 10% formaldehyde and stained with oil red O for the detection of adipocytes.

### Chemotaxis assay

For the chemotaxis assay, 100 μl of SKOV3.PX1 cells (5 × 10^5^ cells/mL in a serum-free medium) were added to the upper decks of the transwells 600 μl condition medium of SKOV3.PX1 and SKOV3.PX1_133^+^44^+^ cells (two cells respectively were cultured with serum-free medium for 24 h as the condition medium) was added to the lower decks. It was observed that the SKOV3.PX1 cells migrated from the upper deck to the lower deck over a 1–3-h period. The movement across the transwell membrane was observed at 1, 2, and 3 h.

### Surface marker expression detected by flow cytometry

Cells were harvested and washed with phosphate buffered saline (PBS) containing 2% FBS from flasks, centrifuged at 300 g at 4°C for 5 min and the cell pellets were collected. We adjusted the cell density to 2.5–3 × 10^6^ cells per assay for the flow cytometry assay. For the cells labeled with fluorochrome antibodies, the experimental procedures followed a standard protocol. Finally, cell pellets were added to a fixation buffer (BD) 100-μl stand at 4°C for 20 min, and then stored at 4°C without light until flow cytometry analysis (BD). Viable cells were identified using the CellQuest software, and the data are shown as logarithmic histograms.

### RNA isolation and reverse transcription–polymerase chain reaction (RT-PCR)

Total RNAs were extracted using TRIzol solution (Invitrogen). cDNA was prepared by incubation of a mixture of 20 μl at 37°C for 1 hour, which contained 2 μg total RNA, 1 μl Moloney murine leukemia virus reverse transcriptase (Promega, Madison, WI), 1 μl deoxyribonucleotide triphosphate, 1 μl oligo-dT and 1 U RNase recombinant RNase inhibitor. Polymerase chain reaction (PCR) was performed in 20 μl of a reaction mixture containing 400 ng cDNA and 500 nM each of *nanog* (sense: 5′- CAAAGGCAAAC AACCCACTT-3′ and antisense: 5′- TCTGCTGGAGGCTGAGGTAT-3′), or beta-actin (sense: 5′ -AGGCGGACTATGACTTAGTTGCGTTACACC-3′ and antisense: 5′- AAGTC CTCGGCCACATTGTGAACTTTG −3′) primers. The PCR products were quantified using a 2% (w/v) agarose gel electrophoresis that was stained with 0.015% RedSafe (iNtRON).

### IHC staining

Paraffin-embedded sections of malignant ovarian epithelial tumors and normal ovarian tissues were stained for CD44 and HER2. These tissue sections of normal ovaries and ovarian serous cystadenocarcinomas were obtained from Pantomics (GenDiscovery Biotechnology, tissue serial no. OVC2281). Briefly, after the paraffin was removed by xylene, the sections were rehydrated in a series of ethanol solutions with decreasing alcohol concentrations, and then washed in PBS. Sections were incubated with the primary antibody for 1 to 2 hours, washed with TBST, and incubated with horseradish peroxidase-conjugated secondary antibodies for 1 hour. Following this, sections were counterstained with hematoxylin. Images of the sections were taken using a microscope equipped with standard optics, and were digitalized. Immunostaining was scored by at levels 1 to 3 for CD44 and CD133 expressions according to the commercial Hercep Test scoring system [19].

### Statistical analyses

All data are shown as mean ± SEM from three independent experiments. The statistical significance of the data was determined using Student's *t*-test, *P*-values < 0.05 were recognized as significant and marked with ‘*’.

## SUPPLEMENTARY MATERIALS



## Abbreviations

ALDH: aldehyde dehydrogenase
ARS: Alizarin Red S
CDH1, CD324: Cadherin-1
CSCs: cancer stem cells
CSLC: cancer stem-like cells
CD: cluster-of differentiation
*CDKN2A*: cyclin-dependent kinase inhibitor 2A
DMSO: dimethyl sulfoxide
MTT: 3-(4,5-dimethylthiazol-2-yl)-2,5-diphenyl- tetrazolium bromide
DTX: diphtheria toxin
EGFR: epidermal growth factor receptor
epithelial cadherin: (E-cadherin uvomorulin)
EpCAM: epithelial cells adhesion molecule
FBS: fetal bovine serum
IHC: immunohistochemistry
Integrin beta 4: (ITGB4 CD104)
IRAK: Interleukin-1 receptor-associated kinase
IBMX: 3-isobutyl-1-methylxanthine
MDR: multi-drug resistance
*MLH1*: mutL homolog 1
MYD88: myeloid differentiation primary response gene 88
*PIK3CA*: phosphatidylinositol-4,5-bisphosphate 3-kinase catalytic subunit alpha
RT-PCR: reverse transcription–polymerase chain reaction
TNF: tumor necrosis factor
TRAF3: tumor necrosis factor -associated factor 3
*TP53*: tumor protein p53
