# Supplementary material for: A rational approach for cancer stem-like cell isolation and characterization using CD44 and prominin-1(CD133) as selection markers
Source: Oncotarget. 2016 Sep 17;7(48):78499–515. doi: 10.18632/oncotarget.12100 (PMC5346656; doi:10.18632/oncotarget.12100)
Supplement: Supplementary file 1 [file oncotarget-07-78499-s001.pdf]

A rational approach for cancer stem-like cell isolation and characterization using CD44 and prominin-1(CD133) as selection markers

Supplementary Materials

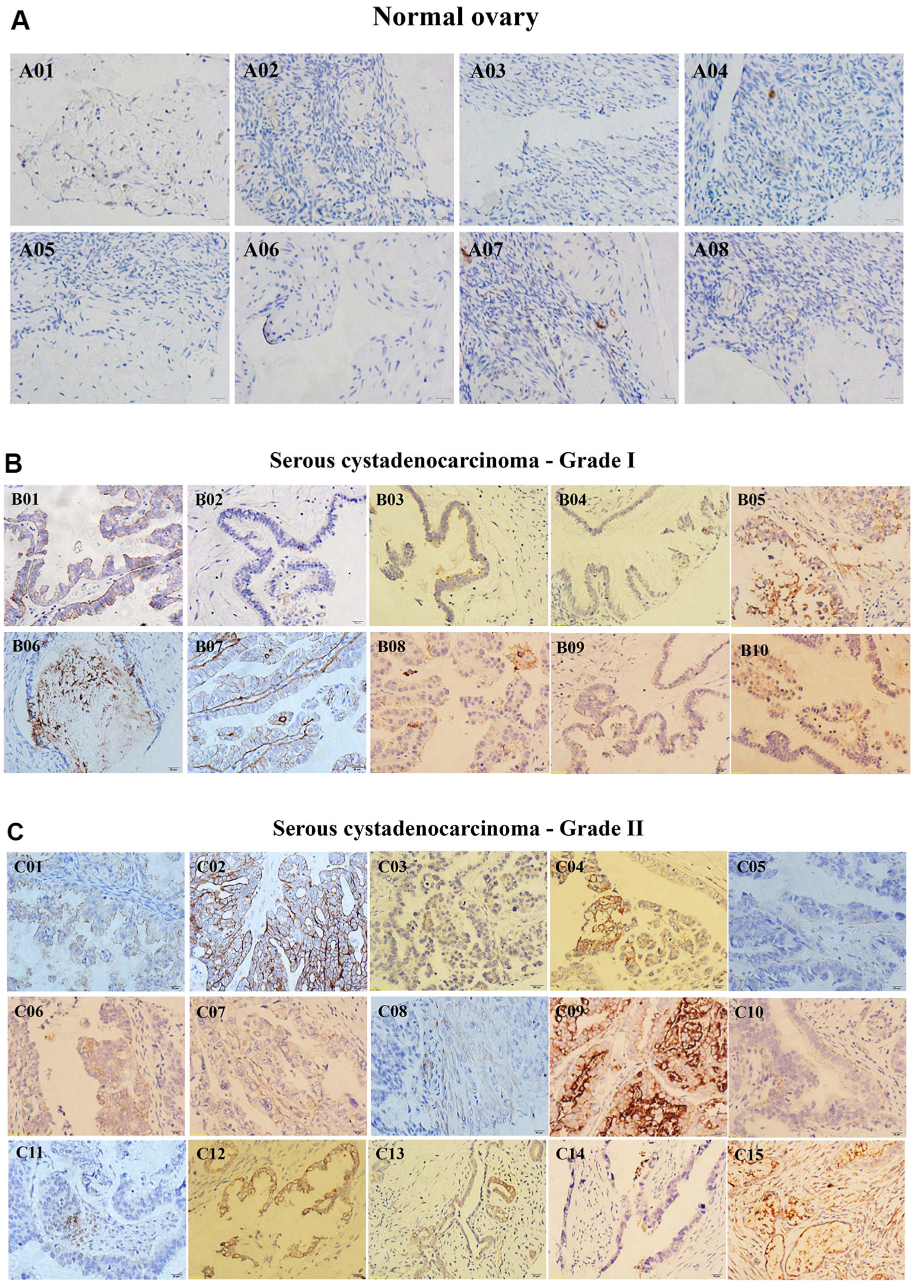

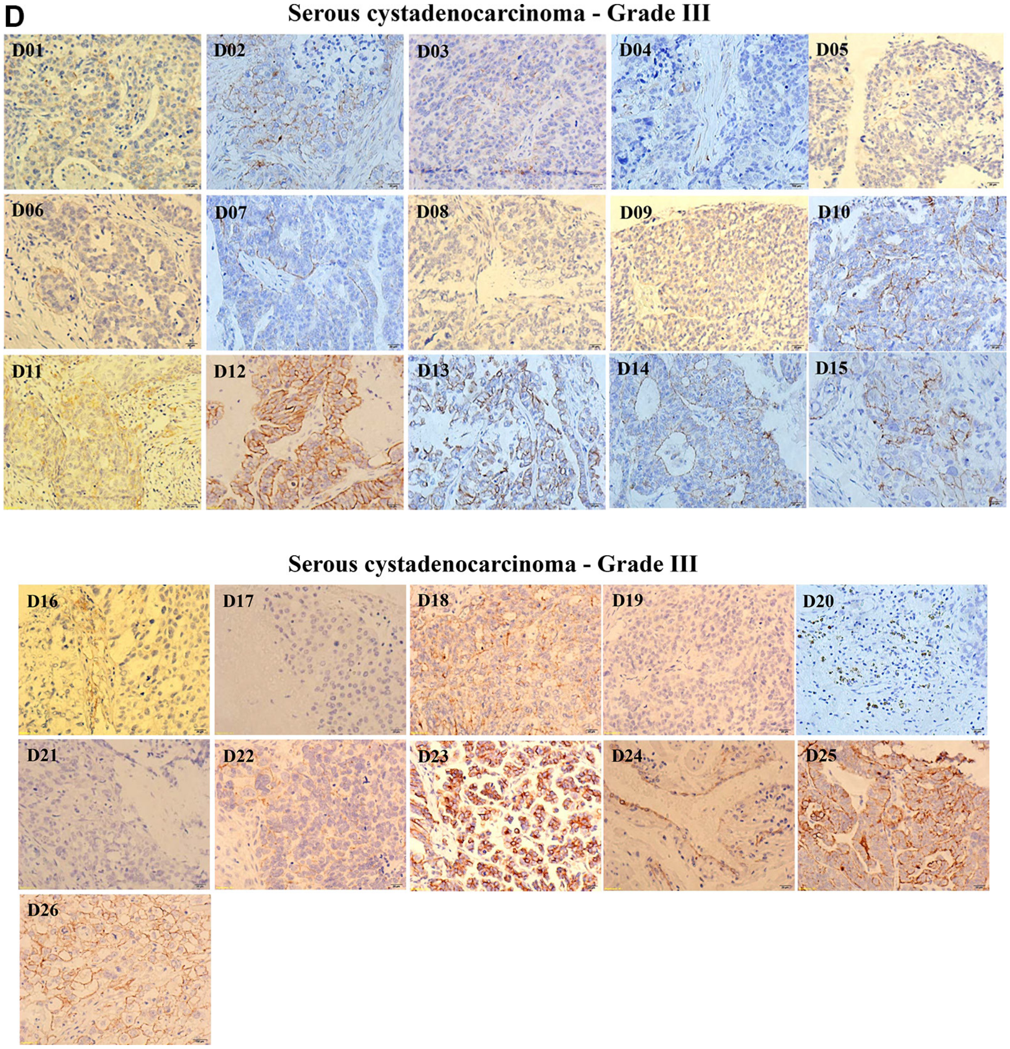

Supplementary Data S1: CD133 expression in normal ovarian tissue and grade-I–III ovarian serous cystadenocarcinomas. (**A**) Immunostaining reveals low levels of CD133 expression in normal ovarian tissue. (**B**) Serous cystadenocarcinoma, grade I; 400× magnification. CD133 immunostaining of ovarian serous cystadenocarcinoma, grade I yielded a score of 1 in 90% of the analyzed samples. (**C**) Serous cystadenocarcinoma, grade II; 400× magnification. (**D**) Serous cystadenocarcinoma, grade III; 400× magnification. Scores of 1–3 were observed. CD133 immunostaining was scored according to the commercial Hercep Test scoring system.
